# Evolution of growth in Gulf of St Lawrence cod?

**DOI:** 10.1098/rspb.2007.1429

**Published:** 2008-02-26

**Authors:** Mikko Heino, Loïc Baulier, David S Boukal, Erin S Dunlop, Sigrunn Eliassen, Katja Enberg, Christian Jørgensen, Øystein Varpe

**Affiliations:** 1 Institute of Marine ResearchPO Box 1870, Nordnes, 5817 Bergen, Norway; 2 Department of Biology, University of BergenPO Box 7800, 5020 Bergen, Norway; 3 International Institute for Applied Systems Analysis2361 Laxenburg, Austria; 4 The University Centre in SvalbardPO Box 156, 9171 Longyearbyen, Norway

Fishing is often size selective such that the likelihood of capture increases with body size. It has therefore been postulated that fishing could favour evolution of slower growth because smaller size would reduce exposure to fishing gear (e.g. Ricker 1981). A recent study by Swain *et al*. (2007; hereafter referred to as SSH) makes a valuable attempt to demonstrate such an effect on length-at-age of southern Gulf of St Lawrence cod (*Gadus morhua*). The strength of their study lies in an innovative combination of three elements. First, as the evolving trait, they used length-at-age 4 years, an age at which cod are representatively sampled but have experienced little fishing mortality. Confounding demographic effects of size-selective fishing were therefore avoided. Second, they had time series of temperature and population density, both possibly affecting length-at-age through phenotypic plasticity. Finally, and as the most innovative element, they linked their approach to quantitative genetics theory. Using a modified breeder's equation, they modelled changes in length-at-age 4 as a function of genetic and environmental components: Δ*L*_4_=*h*^2^*S*+*β*Δ*E*+*ϵ*. Here Δ*L*_4_ and Δ*E* are differences in length-at-age 4 and environment, respectively, between the focal cohort and its parent generation. *S* is the selection differential (difference in mean length-at-age 4 between fish observed at age 4 and those observed at reproducing ages). Estimated heritability *h*^2^ and parameter *β* are regression coefficients, and *ϵ* is a normally distributed error term with zero mean. SSH assumed that the environment can be described by changes in population density Δ*d* and temperature Δ*t*. The key point is that a significant effect of *S* on Δ*L*_4_ would indicate an evolutionary response in length-at-age 4.

SSH's statistically favoured regression model was one including both *S* and Δ*d*; they concluded that the data suggested an evolutionary response to fishing. Of course, as SSH readily pointed out, one cannot rule out the existence of alternative and untested factors. Here, we comment on some caveats in the analysis by SSH. We do not challenge their novel approach, but question some key assumptions and the strength of their conclusions.

## 1. Length-at-age is influenced by reproduction

SSH analysed changes in the mean length of 4-year-old cod and concluded that their results support the hypothesis of a genetic decline in growth. The transition from length-at-age to growth is, however, non-trivial. An individual's length-at-age depends on the environment and at least three life-history traits: growth capacity, maturation schedule and reproductive investment. The *growth capacity* reflects an individual's propensity to forage and the efficiency with which it turns food into body mass. Growth capacity is a quantitative trait under genetic control that can respond to harvest-induced selection (Conover & Munch 2002). Individuals with different growth capacities would have different length-at-age trajectories before maturation even in the same environment. After *maturation*, growth slows down or even stops, because *reproductive investment* channels energy away from growth. Length-at-age measured at or after first reproduction hence depends on all three traits. Only changes in growth capacity equate to what is strictly meant by evolving growth rate.

Data on southern Gulf of St Lawrence cod reveal that from 1990 to 1995, 35–60% of males and 10–50% females were mature at age 4; maturation data outside this 6-year window are unfortunately unreliable and cannot unravel temporal trends (Trippel *et al.* 1997). To reduce the confounding effects of changes in reproductive investment or the proportion of mature fish, we thus recommend that lengths-at-ages before maturation be used as the evolving trait. For southern Gulf of St Lawrence cod, age 3 data are available for the entire time series (Sinclair *et al.* 2002), and the lower proportion of mature individuals at that age would reduce the confounding effects of reproduction.

## 2. Lack of intercept is not trivial

None of the regression models considered by SSH included an intercept. The logic is that if the environment does not change and the selection differential is zero, Δ*L*_4_ will not change and the intercept should be zero. Hidden assumptions are that all relevant environmental variables are included, the data are unbiased and Δ*L*_4_ is genetically uncorrelated with other traits under selection.

To test whether these assumptions hold, we added an intercept to SSH's favoured model (Δ*d*+*S*); it was significantly different from zero (−0.98; *p*=0.03) and *S* became insignificant (*p*=0.34). A model with Δ*d* and an intercept *C* also has a lower AIC value than SSH's favoured model, and *S* is not significant for any other combination of variables. This challenges the conclusion of SSH that the selection differential *S* was driving the change in length-at-age 4. We argue that a significant *C* suggests a negative component to change in length that cannot be statistically ascribed to any of the three explanatory variables.

## 3. Data make robust statistical inference difficult

**Figure 1:**
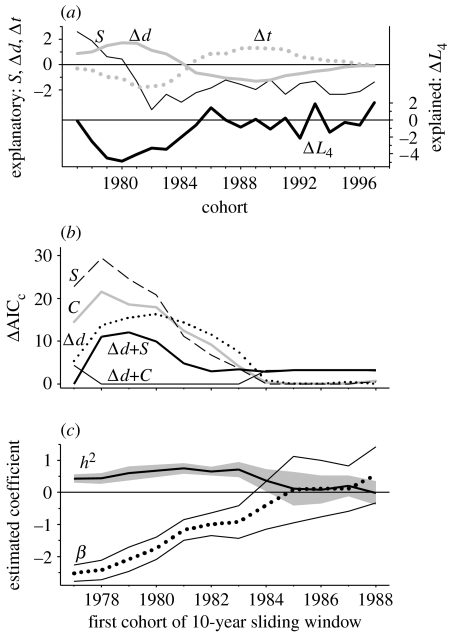
(*a*) Data from fig. 3 in Swain *et al.* (2007). (*b*) Ranking of models involving combinations of selection differential (*S*), change in density (Δ*d*) and intercept (*C*) as explanatory variable(s) in sliding windows of 10 cohorts. ΔAIC_c_ is a small sample version of Akaike's information criterion of each model compared to the best model (the model with the lowest AIC_c_ of all the models considered). The null model, including only an intercept, is shown with a grey line. (*c*) Estimated regression coefficients in sliding windows of 10 cohorts (with 95% CIs) for selection differential and density effects in model 2 (Δ*L*_4_=*β*Δ*d*+*h*^2^*S*) of Swain *et al.* (2007).

Our last point relates to the nature of the data that were available to SSH. The first and second half of the time series of *S* and Δ*L*_4_ differ qualitatively (figure 1*a*). To check robustness of the results presented by SSH, we estimated a range of alternative models for sliding windows of 10 successive cohorts (figure 1*b*; Δ*d* and Δ*t* are strongly correlated and we present only Δ*d* as the results are similar). In the first sliding windows, a range of models can explain the data well (figure 1*b*). Ranked with AIC, SSH's favoured model is among the two best models, but only for the first window does it outperform the model where *S* is replaced by *C*. From the window beginning with cohort 1984, all models become non-significant and the observed change in length can best be interpreted as noise. At the same time, the estimated heritability dropped from between 0.5 and 0.7 to approximately 0 (figure 1*c*), which could be due to the erosion of additive genetic variance. However, it seems unlikely that such high levels of heritability could be purged in one to two cohorts and it does not explain why explanatory variables other than *S* lose significance at the same time (figure 1*b*).

These patterns illustrate how robust differentiation between alternative explanations is compromised when explanatory variables lack contrasts. The difficulty of partitioning statistical effects between correlated variables is well known. However, another difficulty is more specific to the present study, but may pose similar challenges to other studies of directional selection. This relates back to the question of whether to include an intercept. In a model without *C*, any patterns in the data must be ascribed to the explanatory variables of the model, or they end up in the residuals. Here it happens that the pattern is mostly absorbed by *S.* For much of the time series, *S* varies little and is always negative (figure 1*a*); *S* or *C* will therefore have similar effects. As for any explanatory variable, one would wish *S* to show more pronounced patterns, but as this is not the case, the problem appears statistically unresolvable.

## 4. Concluding Remarks

The new approach of Swain *et al.* (2007) holds promise because it simultaneously accounts for the effects of the environment and selection on length-at-age. We look forward to seeing their methodology applied to other fish populations. However, it remains inconclusive as to whether fisheries have induced evolution of reduced growth capacity in the Gulf of St Lawrence cod. Nevertheless, there are changes in length-at-age that cannot be explained by the considered environmental variables. To determine the role of *S*, one would have to assess and control for other factors, such as observation error or unaccounted environmental trends. Neither *S* nor Δ*L*_4_ is directly observable; both are based on model-derived quantities and merge many sources of information. At the same time, the Gulf of St Lawrence has undergone large environmental changes, the effects of which might not be captured by density and temperature alone.
